# COVID-19 Crisis in Jordan: Response, Scenarios, Strategies, and Recommendations

**DOI:** 10.2196/19332

**Published:** 2020-07-07

**Authors:** Raeda Alqutob, Mohannad Al Nsour, Mohammed Rasoul Tarawneh, Musa Ajlouni, Yousef Khader, Ibrahim Aqel, Saad Kharabsheh, Nathir Obeidat

**Affiliations:** 1 Department of Internal Medicine Jordan University Amman Jordan; 2 Global Health Development/Eastern Mediterranean Public Health Network Amman Jordan; 3 Jordan Public Health Forum Amman Jordan; 4 Department of Public Health Jordan University of Science and Technology Irbid Jordan; 5 The Institute for Family Health King Hussein Foundation Amman Jordan

**Keywords:** infection, prevention, public health, pandemic, Jordan, virus, COVID-19

## Abstract

As of April 12, 2020, a total of 389 cases of coronavirus disease were confirmed in Jordan. To control this imminent threat, Jordan has enforced public health infection prevention and control measures, called for social distancing, seized all forms of inbound and outbound movement and international travel, and enacted the Defence Law that transferred the authority to the Minister of Defence to work and formulate orders according to the situation. In an effort to support the government in anticipating the requirements of the health system in the upcoming period, an in-depth reflection and examination of different scenarios of the disease spread were developed. This viewpoint suggests different strategies and measures for case detection and contact tracing, clinical management of cases, public health system functioning, and civil society organizations’ contribution. It is necessary to accelerate containment of the disease to protect the economy and to maintain the continuity of some activities to mitigate the subsequent social, economic, and financial impacts. This requires finding a coping mechanism for a period that may be prolonged until laboratories develop a vaccine. Specifically, it is strongly recommended to promote community health awareness toward public health prevention and control measures, increase the efficiency and comprehensiveness of the epidemiological investigation and active and passive surveillance, and employ technology and digital health solutions to track cases and contacts. It is also recommended to increase and expand resources of intensive care units including respirators, increase the capacity and the number of trained health staff in the area of public health and epidemiology, ensure continued provision of essential public health programs, and mobilize the resources of nongovernmental sectors and donors to provide services for refugees and vulnerable populations.

## Introduction

As of April 13, 2020, more than 1.85 million people are confirmed to have coronavirus disease (COVID-19). Although around 429,028 cases are already recovered, the death toll reached over 114,331 worldwide [1]. Most countries recorded variable rates of COVID-19 cases and deaths, instigating a significant burden on their health systems. As a result, some of these national health systems collapsed, lost control, and became unable to provide health services for a large number of COVID-19 cases and others in need.

## Jordan’s Response to the COVID-19 Pandemic

**Figure 1:**
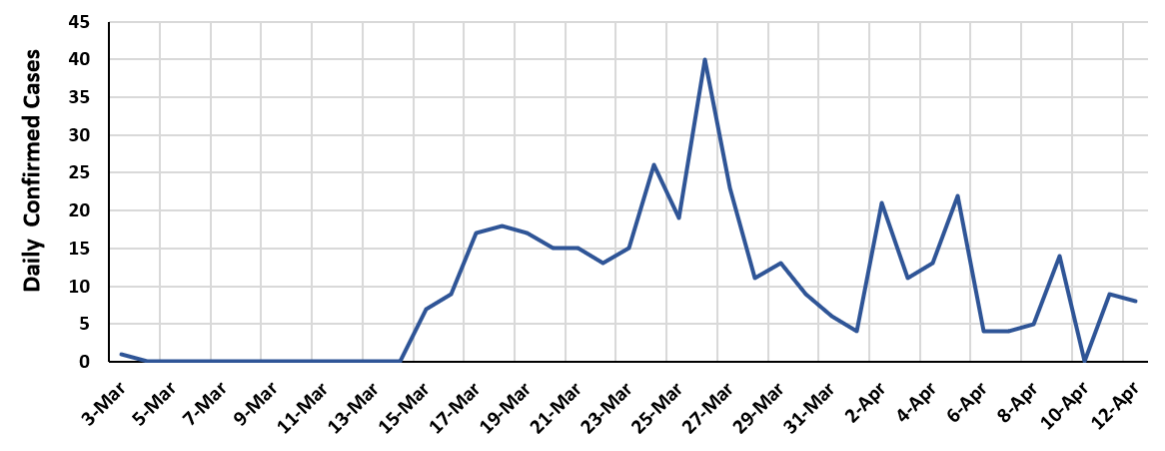
The number of daily confirmed coronavirus disease cases as of April 12, 2020.

According to Jordan’s Ministry of Health data, a total of 389 cases were confirmed across the country as of April 12, 2020. Figure 1 shows the number of daily confirmed COVID-19 cases as of April 12, 2020. The first confirmed case was reported on March 3. However, starting from March 15, the number of cases increased suddenly to 8 cases, and it has been on the rise since then [2]. According to the World Health Organization (WHO) Situation Report 83 released on April 12, 2020, Jordan was classified with a “cluster of cases” transmission for the virus [3]. To control this imminent threat, Jordan has enforced public health infection prevention and control measures. As of March 17, 2020, the government called for social distancing, seized all forms of inbound and outbound movement or international travel, and enacted the Defence Law that transferred the authority to the Minister of Defence to work and formulate orders according to the situation [4]. Consequently, a national curfew was ordered to ensure complete country isolation. It also ordered a lockdown on all border arrivals to the country before March 17 from pandemic countries, and administrative governorates were isolated from each other.

Before embarking on activating the Defense Law, different media channels were used to alert citizens of the seriousness of the virus and its rapid spread. Social media in particular was used heavily in spreading the news about the danger of the disease and groups at high risk of the disease. People were made more aware of the need for social distancing and the importance of personal protection measures. Children and people older than 60 years were the two groups that were specifically addressed by the awareness messages. They were under strict stay-at-home measures, and their care takers were not allowed to accompany them outside of the home except for emergency cases.

Religious leaders, educators, public figures, and opinion leaders were all heavily involved in educating people about the importance of social distancing and infection prevention measures. In addition, the government provided the community, through different channels, with health awareness messages calling for the prevention of the disease and adaptation of healthy behaviors that protect individuals in different social settings. Health awareness was made possible through the Ministry of Health, local and international nongovernmental organizations (NGOs), and academic and research centers. All possible media channels were used including public and private mass communication channels, social media, and religious institutions’ preachers [5].

In Jordan, people still value the family and place emphasis on people’s strong social relationships. This was used to encourage people and families to enforce social distancing and protection measures not only to save their own lives but also to take care of older adults who live within the families and to protect their beloved ones and those who have comorbidities. Mothers who had to stay at home were heavily involved in facilitating online education of their kids, which made it easier for the government to ensure that curfew measures were not affecting child education.

Confirmed and suspected COVID-19 cases from airport arrivals by March 17, 2020, were isolated in hospitals under strict supervision of qualified medical staff. Moreover, the government immediately took measures to ensure preparedness of the health sector. Instantly, the needed equipment and supplies for diagnosis were ordered under the disposal of the National Crises Management Center. Vigorous efforts were exerted to detect and keep track of cases and contacts by outbreak surveillance teams at the national level to contain the spread of the virus and to isolate the cases. The ultimate goal of Jordan was to flatten the disease spread curve to increase the capacity of the health system to absorb new cases.

## Projections of COVID-19 Cases in Jordan

**Figure 2:**
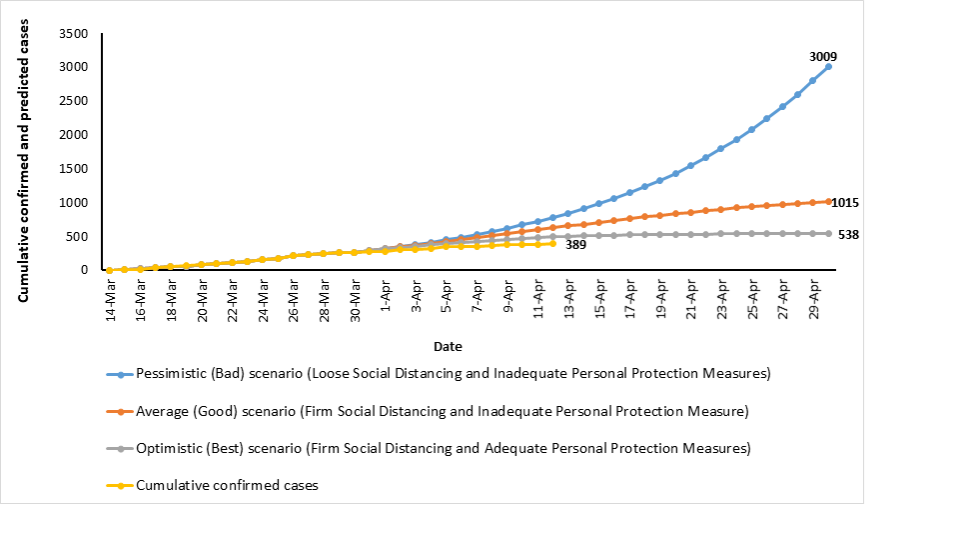
The cumulative confirmed coronavirus disease cases in Jordan and predicted coronavirus disease cases under different scenarios (March 14-April 29, 2020).

About 1 month after the first confirmed case, prolonged national and community-based policies such as isolation, curfews, and social distancing had social and economic implications on individuals and the society at large. In an effort to support the government in anticipating the requirements of the health system in the upcoming period, an in-depth reflection and examination of different scenarios of the disease incidence and spread were developed (Figure 2).

Exponential growth was used for modeling the COVID-19 outbreak in Jordan because epidemiologists have studied those types of outbreaks, and it is well-known that the first period of an epidemic follows exponential growth.

The following exponential growth function (equation 1) was used to predict the number of cases at any given time.

x(t) = *x*_0 *_b^t^**(1)**

In this equation, x(t) is the number of cases at any given time t; *x*_0_ is the number of cases at the beginning, also called the initial value; and *b* is the number of people infected by each sick person, or the growth factor.

The first step was to find the real growth factor of the epidemic, by looking at the data from the epidemic spread in Jordan from March 14-30, 2020, after shifting the first case detected to day 0 (March 14). We used a linear regression after logarithmic transformation of the number of cases and then transformed log(*b*) to “*b*” by applying the exponential (equation 2). The calculated growth factor (*b*)=1.074 and *x*_0_=7.313. “*t*” is the given time.


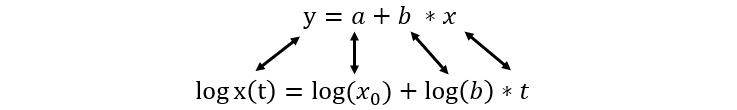
 **(2)**

The number of predicted cases was calculated for each given time (date) using equation 1 under three different scenarios:

- Pessimistic (bad) scenario (loose social distancing and inadequate personal protection measures): we used the growth factor 1.074 based on the number of reported cases.
- Average (good) scenario (strict social distancing and inadequate personal protection measure): we used a growth factor that is decreasing gradually.
- Optimistic (best) scenario (strict social distancing and adequate personal protection measures): we used a growth factor that is decreasing gradually but at a higher rate compared to the good scenario.

In the interpretation of our projection, one should consider that, although the linear model is the best estimate of the exponential growth function, it has a certain error margin. Moreover, the exponential growth function is not necessarily the perfect representation of the epidemic. The exponential growth will only fit the epidemic at the beginning. At some point, cured people will not spread the virus anymore, and when (almost) everyone is or has been infected, the growth will stop. In addition, one should also consider that the hot spots decrease the predictive power of the model.

Based on these predictions, the requirements to strengthen the health system to adapt to the growing needs and the expected contribution of different players in the system were developed.

## Proposed Strategies to Control COVID-19 in Jordan

### Case Detection and Contact Tracing

Considering that the country might fall under the (good) scenario where a predicted total cumulative number of cases will reach 1015 by the end of April 2020, all new cases should be timely diagnosed, and all contacts should be actively traced. This requires a high number of public health specialists or epidemiologists and technicians, an adequate number of laboratory diagnostic tests, and a ready-trained staff to take swaps in-line with standard international practices. Failure to implement this strategy will result in a sharp increase in the number of cases. However, this strategy will place a high burden on the limited number of competent field epidemiologists and on laboratories to carry out tests for cases and potential contacts. To facilitate contact tracing, the government needs to consider the use of information technology and digital initiatives to determine high-spot areas for a better reach of the contacts. Moreover, the government should use the available data on cases and contacts to identify the high-risk geographical areas and high-risk groups to be able to predict the needed resources including intensive care beds and respirators. According to the estimates of the Higher Population Council and considering that around 20% of the population might be symptomatic when affected, 21,684 people are predicted to need hospital care [6]. This will place an unusual burden on the entire health system, which, at best, currently has a hospital bed capacity of 14,701 beds in all health sectors’ service providers. Therefore, it is important that the country secure sufficient numbers of filed workers for case screening and adequate testing kits and laboratory equipment to prevent the overload of the hospital beds. In addition, there will be a need for trained staff to collect and analyze the data on cases, disseminate the data, and share the findings with policy makers for proper decision making, as well as with the local community to ensure transparency. In coordination with the WHO, standardized COVID-19 prevention and control training and implementation guidelines are being developed for health staff and institutions. Further development of online training needs to be put in place.

### Clinical Management of Cases

If the good scenario applies, the confirmed cases must be transferred to the hospital (secondary care), isolated, and provided with appropriate medical care. Although about 25% of COVID-19 cases are either asymptomatic or mildly symptomatic, symptomatic cases must be provided with several hospital care services by specialized doctors based on the specific condition of the patient. Moreover, there is a special need for:

- Availability of proper human resources such as intensive care unit (ICU) nurses, respiratory therapists, radiologic technicians, laboratory technicians, and microbiologists
- Human resource training: all hospital health staff need to undergo special practice training and be aware of how to protect themselves and others from COVID-19. The training must be consistent with international best practices for service provision, infection control, quality assurance, and personal protection inside the hospital and in ambulances.
- Hospital infrastructure preparation: all hospitals need to secure sufficient numbers of hospital beds, isolation rooms, ICU rooms, ventilators, computed topography scans, and secure medical supplies including supplies for sterilization, infection control, and disinfectants inside and outside the hospital environment.
- Appropriate technology and medicines: appropriate medications (currently on the list of nationally approved treatments) to treat and relieve symptoms, appropriate nutrition to boost immunity, and psychological support are all requirements that should be secured.

This strategy requires a lot of human and material resources. The need for these arrangements is substantial if social distancing has not been strictly practiced and personal protection policies and procedures have not been adequately applied. In this case, it is expected that Jordan will be in need of triple the amount of resources or more to meet the need of the 3009 cumulative total number of cases predicted to take place by the end of April under the pessimistic (bad) scenario.

The government should pay attention to the fact that the optimistic scenario is a somewhat comfortable state where social distancing is practiced and personal protection is applied, albeit not totally. This would result in a relatively small increase in the number of cases at the end of April. The government can then deal with the situation effectively and efficiently.

### Public Health System Functioning

The government should use the services of public primary health care centers (PHCs), which are mostly run by the Ministry of Health, by ensuring that centers’ staff are timely and properly assigned to the centers’ program of work. These centers must continue to provide the essential preventive care to children and pregnant women; immunization; screening programs for newborns; family planning services, communicable disease treatment, and surveillance; and mental health services. It is particularly important also to continue to provide paramedic and emergency services in the health centers. This reduces the load on the hospitals at a time when hospital beds and staff are being prepared for any COVID-19 cases. These arrangements should be complemented by strict measures of infection prevention and control at the PHCs. Moreover, staff should also be able to educate patients on protecting themselves against the virus. Accordingly, there should be personal protection protocols for PHCs’ staff members. Care should be provided in a safe context where social distancing is implemented when patients are received, examined, or referred to secondary care (if needed).

The previously mentioned strategies would need a lot of resources for a high-middle income country, which already suffers from a general budget deficit. Within these budget restrictions, the country could develop its partnership with the private sector and give them incentives to possibly modify their production lines such as shifting from garment production to produce masks and needed protection gowns and equipment.

### Civil Society Organizations Contributions

The civil society in Jordan includes NGOs and local community-based organizations (CBOs). NGOs are larger in terms of staff, resources, and financing and operate at a national level with wide geographical coverage. NGOs also have strong systems of governance and accountability. CBOs serve local communities in the geographic areas where they operate and are smaller in terms of staff, resources, and funding, and do not have clear systems of governance and accountability. Therefore, the roles of civil society with all its components must be activated in a phasic approach so that it supports government efforts in addressing this pandemic. Their roles include raising awareness and sustaining delivery of health services; protection; water, sanitation, and hygiene; and social protection services especially in disadvantaged areas as well as marginalized and vulnerable populations including the refugee camps. Civil society can actively contribute to the promotion and activation of social distancing mechanisms and the application of personal protection policy. They can also contribute to facilitating community access and participation. In addition, NGOs can build the capacity of CBOs, unions, political parties, and other community actors on social distancing mechanisms and personal protection policy; provide protection and sterilization kits for staff and clients; and contribute to monitoring cases and contacts, thus supporting the government efforts at local levels.

## Recommendations

There is currently an urgent need to control the COVID-19 crisis and protect the economy at the same time. It is necessary to accelerate containment of the disease to protect the economy and to maintain the continuity of some activities to mitigate the subsequent social, economic, and financial impacts. This requires finding a coping mechanism for a period that may be prolonged until laboratories develop a vaccine. Specific recommendations include the following:

- Promote community health awareness toward the best practices of infection prevention and control and other public health measures such as social distancing
- Increase the efficiency and comprehensiveness of the epidemiological investigation or active and passive surveillance
- Employ technology and digital health solutions to track cases and contacts
- Increase and expand resources of ICUs and respirators
- Increase the number of well-trained, specialized, and supportive health personnel
- Increase the capacity and the number of trained health staff in the area of public health and epidemiology
- Develop and disseminate the guidelines and protocols on the best medical practices in the context of COVID-19
- Train health care professionals in hospitals and primary health centers on infection prevention and control measures
- Ensure continued provision of essential public health programs including vaccination, newborn screening tests, family planning services, communicable disease prevention and control, medical services for chronic diseases, and emergency services
- Mobilize the resources of nongovernmental sectors and donors to create awareness and provide services for refugees and vulnerable populations

## Abbreviations

CBO: community-based organization
COVID-19: coronavirus disease
ICU: intensive care unit
NGO: nongovernmental organization
PHC: primary health care center
WHO: World Health Organization
